# Anti-inflammatory effects of Chinese medicinal herbs on cerebral ischemia

**DOI:** 10.1186/1749-8546-6-26

**Published:** 2011-07-09

**Authors:** Shan-Yu Su, Ching-Liang Hsieh

**Affiliations:** 1 Department of Chinese Medicine, China Medical University Hospital, Taichung 40447, Taiwan; 2 Graduate Institute of Acupuncture Science, China Medical University, Taichung 40402, Taiwan; 3 Acupuncture Research Center, China Medical University, Taichung, 40402, Taiwan

## Abstract

**Abstracts:**

Recent studies have demonstrated the importance of anti-inflammation, including cellular immunity, inflammatory mediators, reactive oxygen species, nitric oxide and several transcriptional factors, in the treatment of cerebral ischemia. This article reviews the roles of Chinese medicinal herbs as well as their ingredients in the inflammatory cascade induced by cerebral ischemia. Chinese medicinal herbs exert neuroprotective effects on cerebral ischemia. The effects include inhibiting the activation of microglia, decreasing levels of adhesion molecules such as intracellular adhesion molecule-1, attenuating expression of pro-inflammatory cytokines such as interleukin-1β and tumor necrosis factor-α, reducing inducible nitric oxide synthase and reactive oxygen species, and regulating transcription factors such as nuclear factor-κB.

## Introduction

Activation of multiple inflammatory cascades accounts for the progressing of ischemia stroke [1]. After cerebral ischemia, energy depletion and necrotic neuron death in the local ischemic area start the inflammatory cascades. The reperfusion generates reactive oxygen species (ROS) that induce the production of cytokines and chemokines leading peripheral leukocytes to influx into the cerebral parenchyma and activate endogenous microglia. Then cellular immunity, adhesion molecules, inflammatory mediators, transcriptional factors participate in the inflammatory process.

**Figure 1:**
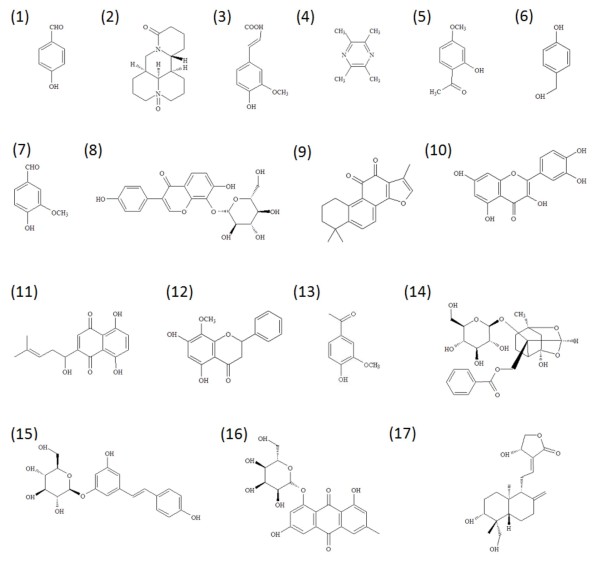
**Chemical structures of active compounds that participates in the inflammatory cascade induced by cerebral ischemia**. (1) 4-hydroxybenzyl aldehyde; (2) oxymatrine; (3) ferulic acid; (4) tetramethylpyrazine; (5) paeonol; (6) 4-hydroxybenzyl alcohol; (7) vanillin; (8) puerarin; (9) tanshinone IIA; (10) quercetin; (11) shikonin; (12) wogonin; (13) apocynin (14) paeoniflorin; (15) polydatin; (16) emodin-8-O-β-D-glucoside; (17) andrographolide.

Anti-inflammatory treatment that inhibits specific steps of the inflammatory cascade is a new strategy for improving outcome after ischemia stroke [2-4]. The anti-inflammatory agents, including a variety of natural products used in Chinese medicine, have been shown to be able to prevent or treat ischemic stroke, by decreasing the infarct area and neurological deficiency [5]. These natural products are documented as anti-oxidative, anti-inflammatory, anti-apoptotic and neuro-functional regulatory agents [5]. Some active ingredients isolated from these herbs have been identified and demonstrated to have neuroprotective actions. Some of these compounds are andrographolide isolated from *Andrographis paniculata *(*Chuan-xin-lian*), oxymatrine isolated from *Sophora flavescens *(*Ku-shen*), quercetin isolated from *Sophora japonica *(*Huai-hua*), ferulic acid isolated from both *Angelica sinensis *(*Dang-gui*) and *Ligusticum wallichii *(*Chuan-xiong*), tetramethylpyrazine isolated from *Ligusticum wallichii *(*Chuan-xiong*), paeonol and paeoniflorin isolated from *Paeonia lactiflora *(*Bai-shao*), shikonin isolated from *Lithospermum erythrorhizon *(*Zi-cao*), vanillin, 4-hydroxybenzyl alcohol and 4-hydroxybenzyl aldehyde isolated from *Gastrodia elata *(*Tain-ma*), puerarin from *Radix Puerariae *(*Pueraria lobata; Ge-gen*), polydatin and emodin-8-O-β-D-glucoside isolated from *Polygonum cuspidatum *(*Hu-zhang*), tanshinone IIA isolated from *Salviae miltiorrhiza *(*Dan-shen*), wogonin isolated from *Scutellaria baicalensis *(*Huang-qin*) and apocynin isolated from *Picrorhiza kurroa *(*Hu-huang-lian*) (Figure 1).

This article reviews the current roles of Chinese medicinal herbs as well as their ingredients in the inflammatory cascade induced by cerebral ischemia. Using cerebral ischemia (OR ischemic stroke) AND herb (OR traditional Chinese medicine) AND inflammation (OR inflammatory OR immunity) as the keywords, we search the English databases including PudMed, Medline, and Cochrane library from 1980 to 2010, generating 77 articles from the initial search.

## Chinese medicinal herbs for reducing inflammation in cerebral ischemia

### Inhibition of cellular immunity

After the onset of ischemia, cellular immunity, including that executed by blood-derived leukocytes, microglia and astrocytes are activated. Immune cells accumulate in the brain tissues, leading to neuronal injury. Leukocytes are the first inflammatory cells recruited into ischemic brain tissues and potentiate injury by secreting deleterious substances and inflammatory mediators [6]. Microglia are activated after ischemia and undergo morphological transformation into phagocytes followed by stimulation of toll-like receptors 4 (TLR-4) [7].

Andrographolide, a diterpenoid lactone isolated from *Andrographis paniculata *that is traditionally used to treat fever [8], reduces the activation of microglia in a cell model of primary rat mesencephalic neuron-glia culture [9]. Apocynin, the main active ingredient of *Picrorhiza kurroa*, blocks microglia activation in a chemical ischemic model of cultured neuroblastoma cells [10] (Table 1). In a rat model of permanent middle cerebral artery occlusion (pMCAo), andrographolide reduces the infarct area at 0.1 mg/kg by reducing the activation of microglia; meanwhile, the inflammation process induced by pMCAo is also suppressed. Paeonol, the active ingredient of *Paeonia lactiflora *traditionally used to treat inflammation-associated allergic rhinitis, otitis and appendicitis, reduces the infarct area and improves the neurological outcome in a transient middle cerebral artery occlusion (tMCAo) rat model by inhibiting the activation of microglia [11]. The aqueous crude extracts of *Sophora japonica*, *Panax notoginseng *and *Zizyphus jujuba *reduce the infarct area in a tMCAo model by modulating cellular immunity. *Sophora japonica*, an anti-oxidative, anti-inflammatory, anti-platelet aggregation and cardiovascular protective agent [12], reduces activated microglia cells labeled by ED1 [13]. *Panax notoginseng*, which is beneficial to the cardiovascular system and is used routinely to treat acute ischemia stroke in China [14] decreases microglical density in the peri-infarct region [15]. *Zizyphus jujuba *protects ischemic damage by decreasing the gliosis of astrocytes and microglia in the CA1 region four days after ischemia/reperfusion [16].

**Table 1:** Medicinal herbs that suppress cellular responses induced by cerebral ischemia


**Targeted cells/molecules**	**Herb or compound**

Microglia/microphage	andrographolide [30], paeonol [11], wogonin [34],*Sophora japonica *[13], *Angelica sinensis *[51], *Panax Notoginseng *[15], apocynin [10]
	
Astrocytes	*Zizyphus jujube *[16]
	
Adhesion molecules	
Selectins	polydatin [24]
Integrins	polydatin [24]
ICAM-1	ferulic acid [26], polydatin [24], *Panax Notoginseng *saponins [22], apocynin [20], paeoniflorin [23]

### Inhibition of adhesion molecules

Adhesion molecules are crucial in the recruiting of leukocytes into the brain parenchyma after ischemia. The interaction between leukocytes and the vascular endothelium is mediated by three main groups of cell adhesion molecules, namely selectin (P-selectin, E-selectin, and L-selectin), the immunoglobulin superfamily including intra-cellular adhesion molecule-1 (ICAM-1), ICAM-2 and vascular cell adhesion molecules-1 (VCAM-1), and integrins [17]. The suppression of adhesion molecules is considered an important therapeutic target [18].

Apocynin, isolated from *Picrorhiza kurroa*, attenuates both cerebral infarct volume and neurological defect in ischemia/reperfusion rat models [10,19-21]. The neuroprotection by apocynin is accompanied by the suppression of ICAM in ischemic regions [20]. Treatment of saponins extracted from *Panax notoginseng *and paeoniflorin from *Paeonia lactiflora *inhibits expression of ICAM-1 and MPO activity in a tMCAo rat model [22,23]. Polydatin. *i.e*. 3,4',5-trihydroxystilbene-3-β-mono-D-glucoside, one of the components isolated from *Polygonum cuspidatum*, protects the brain from leukocyte recruitment after ischemia injury by decreasing adhesion molecules, including ICAM-1, VCAM-1, E-selectin, L-selectin and integrins [24]. *Polygonum cuspidatum *is traditionally used in inflammatory diseases, including dermatitis, abscess and hepatitis [25]. Ferulic acid, the active compound of *Angelica sinensis *and *Ligusticum wallichii*, exhibits similar effects. Intravenous injection of ferulic acid (80 and 100 mg/kg) at the beginning of tMCAo reduces cerebral infarct area and improves neurological functions measured by neurological deficit scores in rats by blocking ICAM-1 activity [26].

### Regulation of cytokines

Pro-inflammatory cytokines drive the inflammatory process and aggravate inflammation. Cytokines that participate in the inflammation after cerebral ischemia include the neurotoxic cytokines interleukin-1β (IL-1β), tumor necrosis factor-alpha (TNF-α), neuroprotective cytokines interleukin-6 (IL-6), interleukin-10 (IL-10) and transforming growth factor-β [27]. Among these cytokines, IL-1 and TNF-α are shown to be decreased by several herbs (Table 2). Total saponins extracted from *Panax notoginseng *reduce IL-1 activity [28]. Paeonol, apocinin and the aqueous extract of *Sophora japonica *reduce IL-1β immune-reactive cells in brain parenchyma of a tMCAo model [13,20,29]. Andrographolide, paeoniflorin and andrographolide inhibit both TNF-α and IL-1β simultaneously [23,30]. Both puerarin, the principal bioactive isoflavonoid derived from peuraria lobata and wogonin (5,7-dihydroxy-8-methoxyflavone) isolated from the root of *Scutellaria baicalensis *exert neuroprotection by inhibiting TNF-α. *Radix puerariae *is a medicinal plant used as antipyretic, antidiarrhetic, diaphoretic and antiemetic agents [31]. Ethanol extract of *Radix puerariae *acts as an anti-depressant in mice undergoing cerebral ischemia/reperfusion [31]. Puerarin reduces infarct volume in the tMCAo rat model at 50 mg/kg. The associated mechanisms include the ability to down-regulate TNF-α [32]. Methanol extracts from the dried roots of *Scutellaria baicalensis *(0.1-10 mg/kg) significantly protect CA1 neuronal cells against transient forebrain ischemia [33]. Wogonin induces TNF-α and protects hippocampal neuron from death in a transient global ischemia by four-vessel occlusion in rats [34].

**Table 2:** Herbs or herbal compounds that suppress the production of inflammatory mediators and transcription factors activated by cerebral ischemia


**Targeted molecules**	**Herb or compound**

Inflammatory meciators	
Cytokines	
IL-1β	andrographolide [30], paeonol [11],*Sophora japonica *[13],*Panax Notoginseng *saponins [28], apocynin [20], *Scutellaria baicalensis *flavonoid [54]
TNF-α	andrographolide [30], puerarin [32], wogonin [34]
	
NO	
iNOS	ferulic acid [38], puerarin [32], tetramethylpyrazine [40], wogonin [34], *Panax Notoginseng *[15]
	
ROS	*Salviae Miltiorrhiza*[41], 4-hydroxybenzyl alcohol [48], *Angelica sinensis *[51], tanshinone IIA [45]
MPO	ferulic acid [26], tetramethylpyrazine [52], *Anemarrhena asphodeloides *[53], *Panax Notoginseng *saponins [22]
SOD	shikonin [57], paeonol [29], emodin-8-O-β-D-glucoside [58], *Zizyphus jujube *[16], *Scutellaria baicalensis *flavonoid [54]
CAT	*Scutellaria baicalensis *isoflavoid [54], shikonin [57]
GSH	shikonin [57]
OH·	tetramethylpyrazine [40]
NADPH	Apocinin [20]
MMP	quercetin [62], *Panax Notoginseng *saponins [64]
Transcription factors	
NF-κB	andrographolide [30], oxymatrine [81], feulic acid [26], paeoniflorin [82], wogonin [80],*Panax Notoginseng *[15], apocynin [20]
P38	Oxymatrine [73], ferulic acid [38]

### Inhibition of oxidative stress and NO

After cerebral ischemia, reperfusion leads to the generation of ROS by several enzymes. Superoxide anion is generated by cyclooxygenase (COX), xanthine dehydrogenase, xanthine oxidase, nicotinamide adenine dinucleotide phosphate (NADPH) oxidase and hypochlorous; hydrogen peroxide (H_2_O_2_) are generated by myeloperoxidase (MPO) and monoamine oxidase (MAO). Among these, superoxide anion reacts with NO to generate peroxynitrite [35]. ROS stimulates ischemic cells to secrete inflammatory cytokines and chemokines which cause adhesion molecule up-regulation in the cerebral vasculature and peripheral leukocyte recruitment. Once activated, inflammatory cells release a variety of cytotoxic agents such as cytokines, matrix metalloproteinases (MMPs), NO and ROS [36]. The MMPs are proteases that break down extracellular proteins such as collagen, leading to extracellular matrix remodeling in the inflammatory response [37]. Among the three isoforms of NOS, namely inducible NOS (iNOS), neuronal NOS (nNOS) and endothelial NOS (eNOS), iNOS expression is restricted to cells involved in inflammatory responses such as circulating leukocytes, microglia, and astrocytes and therefore, iNOS is thought to be the most contributive NOS contributes to the ischemic injury via generating nitric oxide (NO) [36].

Herbs and their ingredients that exert neuroprotective effects *via *inhibiting NO include ferulic acid, puerarin, tetramethylpyrazine, wogonin and *Panax notoginseng*. Intravenous injection of ferulic acid (80 and 100 mg/kg) at the beginning of tMCAo abrogates the elevation of nNOS, iNOS and p38 activation, leading to the decrease of the number of relevant apoptotic cells in the ischemia brain [38]. The inhibition of TNF-α by puerarin is followed by the inhibition of iNOS expression and active caspase-3 formation, resulting in a reduction in the infarct volume in ischemia-reperfusion brain injury [32]. Wogonin reduces iNOS after cerebral ischemia [34]. Tetramethylpyrazine, which is isolated from *Ligusticum wallichiit*, protects brain from ischemia insult [39]*via *decreasing nitrotyrosine, iNOS and hydroxyl radical formation [40].

In a tMCAo rat model, the luminal luciferase count in the brain parenchyma is suppressed by *Salviae miltiorrhiza *[41], which is used as a common herb to treat acute ischemic stroke [42]. Aqueous extract of *Salviae miltiorrhiza *reduces the infarct area and preserves pyramidal cells in tMCAo rats [43] as well as the NOS gene expression in the cerebral cortex and caudate-putamen in the ischemic brain [44]. The active component of *Salviae miltiorrhiza*, tanshinone IIA (10 mg/kg, i.p.), exhibits high anti-oxidative activities in a rat model of hypoxia-ischemia encephalopathy, in which the rat is exposed to a low oxygen environment (8%) and the right common carotid artery is ligated [45]. The same neuronal protective effect exists in the neonatal brain with hypoxia-ischemia injury [45].

Aqueous extract of *Gastrodia elata*, which is widely used to treat convulsive disorders, protects the brain from ischemia in rat and in gerbil models [46,47]. The active compound isolated from *Gastrodia elata*, 4-hydroxybenzyl alcohol, may explain the neuro-protection activity. It increases the antioxidant protein including protein disulfide isomerase and 1-Cys peroxiredoxin (1-Cys Prx) [46]. The down-regulation of 8-hydroxy-2'-deoxyguanosine suggests that 4-hydroxybenzyl alcohol scavenges free radicals [48], which may be related to its inhibition of apoptosis in a rat tMCAo model [49]. Another two compounds isolated from *Gastrodia elata*, namely vanillin and 4-hydroxybenzyl aldehyde, also show neuroprotective ability in cerebral ischemia. Among the three compounds isolated from *Gastrodia elata*, vanillin-treated animals have the greatest neuronal survival after ischemia insult [48]. Treatment of *Angelica sinensis *(5 g/kg) simultaneously with cerebral ischemia reduces the infarct area caused by tMCAo [50]. Oral feeding of aqueous extracts of *Angelica sinensis *for seven days (250 mg/kg/day) attenuates oxidative stress in the brain [51].

Several Chinese medicinal herbs produce their neuroprotective effects *via *suppression of MPO, including ferulic acid, tetramethylpyrazine, *Anemarrhena asphodeloides *and *Panax notoginseng *saponins. At the beginning of tMCAo, intravenous injection of ferulic acid (80 and 100 mg/kg) suppresses the expression of MPO [26]. The protective effects of tetramethylpyrazine and *Panax notoginseng *saponins are associated with the reduced ischemia/reperfusion induced MPO activity levels, indicating that *Panax notoginseng *saponins decreases the production of ROS and ROS-related inflammatory activity [52]. The aqueous extract of *Anemarrhena asphodeloides *increases MPO activity and protects animals from ischemia/reperfusion injury with a therapeutic time window from one hour prior to reperfusion to two hours after reperfusion [53].

Chinese medicinal herbs that suppress ROS by increasing the activity of antioxidative enzymes include *Scutellaria baicalensis *flavonoid, shikonin, paeonol, emodin-8-O-β-D-glucoside and *Zizyphus jujube *extract. In a permanent cerebral ischemic model in rats, in which the bilateral common carotid arteries are ligated, oral feeding of total flavonoid (17.5-70 mg/kg) from *Scutellaria baicalensis *increase SOD and catalase (CAT) activity in the hippocampus and cerebral ischemia cortex [54]. Paeonol as well increases superoxide dismutase (SOD) activity after cerebral ischemia [29]. Shikonin is a naphthoquinone pigment isolated from *Lithospermum erythrorhizon*, which is traditionally used to heal wounds and treat inflammatory dermatological diseases [55,56]. Shikonin protects the brain from ischemia in the tMCAo mouse model by acting as an antioxidant. It up-regulates SOD, catalase, glutathione peroxidase (GSH-Px) activities and down-regulates glutathione (GSH)/glutathione disulfide (GSSG) ratio [57]. Paeonol also exerts anti-oxidative activity by increasing superoxide dismutase (SOD) activity [29]. Emodin-8-O-β-D-glucoside, extracted from *Polygonum cuspidatum*, increases the total antioxidant capacity of cells after cerebral ischemia. Increased SOD level and decreased MDA level reduce infarct area and neurological defect [58]. The anti-inflammatory effects of *Zizyphus jujuba *come from the reduction of hydroxynonenal level, an indicator of lipid peroxidation and elevation the SOD level [16]. Several compounds are isolated from *Zizyphus jujuba*, such as jujuboside [59], triterpenic acid [60] and saponins [61], but the specific active compound responsible for the neuroprotective effects has yet to be identified. As an NADPH oxidase inhibitor, apocinin exerts neuroprotective effects by the blockage of ROS production in leukocytes *via *the inhibition of NADPH oxidase, leading to the elimination of cytokine and adhesion molecule production [20].

Some Chinese medicinal herbs have effects on MMP-9. Quercetin, one of the flovonoids isolated from *Sophora japonica*, protects the blood-brain barrier and elevates MMP-9 levels in the photothrombotic animal model while the level of MMP-2 is not regulated by quercetin [62]. Total saponins extracted from *Panax notoginseng *reduces the expression of caspase-1 and caspae-3, resulting in the attenuation of apoptosis [63]. *Panax notoginseng *saponins reduce protein levels of MMP-9 in a mouse tMCAo model [64]. Three major bioactive saponins have been identified to be ginsenoside Rg_1_, ginsenoside Rb_1 _and notoginsenoside R_1 _[65].

### Regulation of chemokines

Chemokines have a deleterious role by increasing leukocyte infiltration after stroke [66]. Arachidonic acid (AA) cascade is a downstream signal pathway of immune cells initiated *via *phospholipase A2 (PLA2) and phospholipase C (PLC) which is activated by calcium accumulation caused by cessation of energy by ischemia [67]. PLA2 hydrolyzes glycerophospholipids to release AA, which is metabolized through two different pathways *via *cyclooxygenase (COX) or lipoxygenase (LOX). The COX pathway converts AA to prostaglandin H2 (PGH2) which is then metabolized into eicosanoids, including prostacyclin (PGI2), thromboxane A2 (TXA2), prostaglandin E2 (PGE2) and prostaglandin D2 (PGD2) [68]. These eicosanoids affect vasomotor regulation and increase microvascular and blood-brain barrier (BBB) permeability [69,70]. AA is also converted to 5-hydroperoxyeicosateraenoic acid (5-HPETE) by 5-LOX. 5-HPETE is then metabolized to leukotrienes to mediate chemoattraction, brain edema and BBB permeability [71].

**Figure 2:**
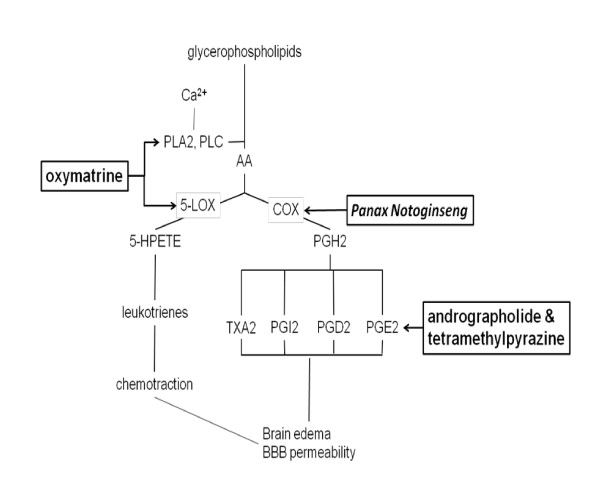
**Molecular targets of herbal medicines for interrupting arachidonic acid metabolism**.

Ingredients from *Sophora flavescens*, *Panax notoginseng*, *Andrographis paniculata*, and *Ligusticum wallichii *block chemokines after cerebral ischemia. *Sophora flavescens *is used for anti-oxidation, anti-bacterial, anti-inflammation, anti-fever, anti-arrhythmia, anti-asthma, anti-ulcer and anti-neoplasm [72]. One of the major alkaloids of *Sophora flavescens*, oxymatrine, reduces the overexpression of phosphorylated p38, 12/15 LOX and cytosolic PLA2 [73]. The alkaloid-free fraction extracted by EtOAc containing two major flavonoids kurarinone (45.5%) and sophoraflavone G (14.7%) protects the brain from injury of pMCAo [74]. The underlying protective mechanisms of *Panax notoginseng *comprise the inhibition COX-2 *via *blocking the nuclear factor-κB (NF-κB) pathway [15]. Andrographolide reduces the infarct area in a rat model of pMCAo by decreasing AA metabolism into PGE [30]. Moreover, tetramethylpyrazine reduces PGE2 levels induced by ischemia/reperfusion [52] (Figure 2).

### Transcription factors

During the inflammatory process, activation of a specific transcription factor, including NF-κB, mitogen-activated protein kinase (MAPK), activator protein-1 (AP-1) and regulation of specific gene expression are needed. Many inflammatory genes contain NF-κB binding site, such as TNF-α, ICAM-1, iNOS and IL-6 [75].

Three MAPKs are documented during cerebral ischemia, namely the stress-activated protein kinases/c-Jun N-terminal kinase (SAPK/JNK), p38 MAPK and the extracellular signal-regulated kinases (ERKs). P38 MAPK stabilizes and enhances the translation of mRNA encoding pro-inflammatory protein [76]. The reduction of ERKs is necessary for the recovery from ischemic stroke [77]. Mediated through JNK cascade, AP-1 is activated by the up-regulation of c-fos 30 minutes after the onset of a stroke [78]. The p38 MAP kinase participates in the mRNA expression of c-jun and c-fos after cerebral ischemia [79].

Several Chinese medicinal herbs block inflammation by inhibiting the NF-κB pathway, including andrographolide, oxymatrine, feulic acid, paeoniflorin, wogonin,*Panax notoginseng *and apocynin [30,80,81]. *Panax notoginseng *inhibits inflammatory mediators, including iNOS and COX-2 by blocking the NF-κB pathway [15]. In a chronic cerebral ischemia rat model, in which bilateral carotid arteries are permanently occluded, paeoniflorin (25 mg/kg) decreases the expression of NF-κB in astrocyte and microglia within hippocampal area [82]. The protective effect provided by wogonin has been demonstrated in a pMCAo model, in which wogonin reduces the total volume of infarction and improves behavior functions [83], associated with the reduction of NF-κB activity, but not with the regulation of mitogen-activated protein kinases family members, p38, ERK and JNK [80]. The inhibition of COX-2 by *Panax notoginseng *may be achieved *via *blocking the NF-κB pathway [15]. Apocynin reduces inflammation also *via *the inhibition of NF-κB [20]. The reduction of LOX, PLA2 and TLR by oxymatrin may be related to the inhibiting of the NF-κB and p38 activation [73]. The decreases of ICAM-1 and MPO by ferulic acid also considered a result of the suppression of NF-κB [26] and the inhibition of p38 may lead to the decrease of relevant apoptosis [38].

### Clinical trials

Most clinical trials of Chinese medicine on ischemic stroke test the efficacy of multi-herb formulae. For example, *Danqi Piantang Jiaonang *containing *Salviae miltiorrhiza*, *Ligusticum wallichii*, *Angelica sinensis *improved neurological recovery in patients after a stroke [84]. A multi-center randomized controlled trial (RCT) suggested *Danqi Piantang Jiaonang *to increase the scores evaluated by diagnostic therapeutic effects of Apoplexy scoring system in post-stroke rehabilitation and in the recovery of patients with posterior circulation infarction and severe ischemic stroke [85,86]. Two other clinical studies for two Chinese herbal formulae, namely *Dengzhan Shengmai *capsule and *Huatuo Zaizao Wan *are currently in progress. On the other hand, few single herbs have been tested in clinical trials. In a multi-center, double-blinded, randomized controlled clinical trial of 140 patients suffering subacute ischemic stroke, *Panax notoginseng *ameliorated neurological deficit and activities of daily living [87]. Chen *et al *reported that by reviewing several papers including 660 patients in RCTs, *Panax notoginseng *was safe and beneficial [14]. *Salviae miltiorrhiza *has been studied in clinical trials; however, the results were inconclusive. A systematic review of 33 *Salviae miltiorrhiza *trials for acute ischemic stroke did not support the efficacy of *Salviae miltiorrhiza *in disability improvement after acute ischemic stroke [42]. These clinical trials share similar problems, *e.g*. lack of placebo-controlled trial and small sample size [14,42].

## Conclusions

Many Chinese medicinal herbs that act on the inflammation process were used to treat ischemia stroke. These herbs suppress inflammatory cascades in cellular immunity, adhesion molecules, cytokines, arachidonic acid, metabolites, NO, ROS, and transcriptional factors. In the future, more clinical trials should be down to Chinese herbs that have been demonstrated effective in animal studies but not been proven in human.

## Abbreviations

AA: arachidonic acid; AP-1: activator protein-1; BBB: blood-brain barrier; COX: cyclooxygenase; 1-Cys Prx: 1-Cys peroxiredoxin; ERKs: extracellular signal-regulated kinase; GSH: glutathione; GSH-Px: glutathione peroxidase; GSSG: glutathione disulfide; 5-HPETE: 5-hydroperoxyeicosateraenoic acid; ICAM-1: intra-cellular adhesion molecule-1; ICAM-2: intra-cellular adhesion molecule-2; IL-1: interleukin-1; IL-6: interleukin-6; IL-10: interleukin-10; JNK: c-Jun N-terminal kinase; LOX: lipoxygenase; MAO: monoamine oxidase; MAPK: mitogen-activated protein kinase; pMCAo: permanent middle cerebral artery occlusion; tMCAo: transient middle cerebral artery occlusion; MPO: myeloperoxidase; MMPs: metalloproteinases; NF-κB: nuclear factor-κB; NADPH: nicotinamide adenine dinucleotide phosphate; NO: nitric oxide; eNOS: endothelial nitric oxide synthase; iNOS: inducible NOS; nNOS: neuronal nitric oxide synthase; PGI2: prostacyclin; PLA2: phospholipase A2; PGD2: prostaglandin D2; PGE2: prostaglandin E2; PLC: phospholipase C; ROS: reactive oxygen species; SAPK: stress-activated protein kinases; SOD: superoxide dismutase; TLR-4: toll-like receptors 4; TNF-α: tumor necrosis factor-α; TXA2: thromboxane A2; VCAM-1: vascular cell adhesion molecules-1.

## Competing interests

The authors declare that they have no competing interests.

## Authors' contributions

CLH designed the study and revised the manuscript. SYS conducted the literature search and drafted the manuscript. Both authors read and approved the final version of the manuscript.
